# Chromosome 9p copy number gains involving PD-L1 are associated with a specific proliferation and immune-modulating gene expression program active across major cancer types

**DOI:** 10.1186/s12920-017-0308-8

**Published:** 2017-12-06

**Authors:** Jan Budczies, Carsten Denkert, Balázs Győrffy, Peter Schirmacher, Albrecht Stenzinger

**Affiliations:** 1 Institute of Pathology, Charité – Universitätsmedizin Berlin, Corporate Member of Freie Universität Berlin, Humboldt-Universität zu Berlin, and Berlin Institute of Health, Berlin, Germany; 2 German Cancer Consortium (DKTK), Berlin and Heidelberg partner sites, and German Cancer Research Center (DKFZ), Heidelberg, Germany; 3 MTA TTK Lendulet Cancer Biomarker Research Group, Budapest, Hungary; 4 2nd Dept. of Pediatrics, Semmelweis University, Budapest, Hungary; 5 Institute of Pathology, University Hospital Heidelberg, Im Neuenheimer Feld 224, 69120 Heidelberg, Germany

**Keywords:** Cancer, Immunotherapy, PD-L1, DNA copy number alterations, Chromosome 9p gain

## Abstract

**Background:**

Inhibition of the PD-L1/PD-1 immune checkpoint axis represents one of the most promising approaches of immunotherapy for various cancer types. However, immune checkpoint inhibition is successful only in subpopulations of patients emphasizing the need for powerful biomarkers that adequately reflect the complex interaction between the tumor and the immune system. Recently, recurrent copy number gains (CNG) in chromosome 9p involving PD-L1 were detected in many cancer types including lung cancer, melanoma, bladder cancer, head and neck cancer, cervical cancer, soft tissue sarcoma, prostate cancer, gastric cancer, ovarian cancer, and triple-negative breast cancer.

**Methods:**

Here, we applied functional genomics to analyze global mRNA expression changes associated with chromosome 9p gains. Using the TCGA data set, we identified a list of 75 genes that were strongly up-regulated in tumors with chromosome 9p gains across many cancer types.

**Results:**

As expected, the gene set was enriched for chromosome 9p and in particular chromosome 9p24 (36 genes and 23 genes). Furthermore, we found enrichment of two expression programs derived from genes within and beyond 9p: one implicated in cell cycle regulation (22 genes) and the other implicated in modulation of the immune system (16 genes). Among these were specific cytokines and chemokines, e.g. CCL4, CCL8, CXCL10, CXCL11, other immunoregulatory genes such as IFN-G and IDO1 as well as highly expressed proliferation-related kinases and genes including PLK1, TTK, MELK and CDC20 that represent potential drug targets.

**Conclusions:**

Collectively, these data shed light on mechanisms of immune escape and stimulation of proliferation in cancer with PD-L1 CNG and highlight additional vulnerabilities that may be therapeutically exploitable.

**Electronic supplementary material:**

The online version of this article (10.1186/s12920-017-0308-8) contains supplementary material, which is available to authorized users.

## Background

Programmed cell death 1 (*PD-1*)/PD-ligand1 (*PD-L1*) checkpoint inhibition has emerged as a promising treatment strategy in different advanced and often otherwise untreatable tumors in a growing number of cancer types. In this context immunohistochemical (IHC) evaluation of *PD-L1* protein expression has been proposed as a biomarker that predicts treatment efficacy. However, different IHC-antibody clones used for *PD-L1* detection and different evaluation methods including different cutoff values to distinguish ‘positive’ from ‘negative’ tumors introduce a substantial variance in the determination of *PD-L1* status. These ambiguities and the complex interplay of cancer-cell-inherent, microenvironment-associated and host-related factors [1] advocate further investigation of the underlying biology to identify additional biomarkers that aid current stratification strategies.

*PD-L1* copy number gains (CNG) are prevalent in significant subsets of different cancers including triple negative breast cancer [2], Hodgkin’s lymphoma [3], cancer of unknown primary [4], NSCLC [5], SCLC [6], and gastric cancer [7]. In particular, Hodgkin’s lymphomas harboring *PD-L1* CNG were reported to exhibit increased *PD-L1* expression and to respond well to immune checkpoint inhibition [3]. Adding to the growing body of evidence, enabled by The Cancer Genome Atlas (TCGA) a recent pan-cancer analysis of almost 10,000 tumors showed that *PD-L1* CNG were abundant in many cancer types with both focal and non-focal gains typically occurring at frequencies between 2% and 10% [8]. In this study, we identified a 7.8-Mbp region of 38 genes located in chromosome 9p24 (core amplified region) that is co-amplified with *PD-L1* in more than 80% of tumors with focal *PD-L1* gains across 22 cancer types. The core amplified region includes the genes *PD-L1*, PD-ligand 2 (*PD-L2*) and Janus kinase 2 (JAK2). Following up on these findings, we here show that chromosome 9p copy number gains are associated with specific mRNA expression changes enriched for genes implicated in cell cycle regulation and immune cell response.

## Methods

*PD-L1* copy number alteration (GISTIC) and genome-wide mRNA expression data (RNAseq v2) of the following 21 cancer types profiled in the TCGA project were obtained from the cBioPortal [9]: Bladder Urothelial Carcinoma (BLCA), Breast invasive carcinoma (BRCA), Cervical squamous cell carcinoma and endocervical adenocarcinoma (CESC), Colorectal adenocarcinoma (COADREAD), Glioblastoma multiforme (GBM), Head and Neck squamous cell carcinoma (HNSC), Kidney renal clear cell carcinoma (KIRC), Kidney Renal Papillary Cell Carcinoma (KIRP), Brain Lower Grade Glioma (LGG), Liver hepatocellular carcinoma (LIHC), Lung adenocarcinoma (LUAD), Lung squamous cell carcinoma (LUSC), Ovarian serous cystadenocarcinoma (OV), pancreatic adenocarcinoma (PAAD), Pheochromocytoma and Paraganglioma (PCPG), Prostate Adenocarcinoma (PRAD), Sarcoma (SARC), Skin Cutaneous Melanoma (SKCM), Stomach adenocarcinoma (STAD), Papillary Thyroid Carcinoma (THCA), and Uterine Corpus Endometrial Carcinoma (UCEC). All TCGA data were freely available without restrictions on their use in publications (https://cancergenome.nih.gov/publications/publicationguidelines).

*PD-L1* copy numbers alterations were evaluated using the calls reported by the TCGA consortium based on Affymetrix SNP 6.0 array data and the GISTIC 2.0 algorithm [10]. *PD-L1* status was assessed as “loss” for calls −2 and −1, as “normal” for call 0, and as “gain” for calls 1 and 2. Copy number calls and upper quartile normalized RSEM estimates of mRNA expression were obtained from the cBioPortal (http://www.cbioportal.org). Expression values were log2(x + 1) transformed prior to statistical analysis and visualization. Differential expression between tumors with and without *PD-L1* gains as well as between tumors with and without *PD-L1* losses was assessed using Welch’s t-test. Hierarchical clustering was performed using Pearson correlations as similarity measure and the average linkage method to determine the distance between clusters. Non-significant fold changes were set to one before clustering. In a genome-wide mRNA expression analysis, a list of 75 strongly and recurrently up-regulated genes was identified by filtering for genes showing significant (*p* < 0.05) and at least 1.5-fold enhanced mRNA expression in tumors with *PD-L1* gains across at least 6 cancer types (Additional file 1: Table S1). The fold change threshold was added to the gene selection criterion to concentrate on strong expression changes that can be potentially therapeutically targeted. Pan-cancer significance was assessed by combining the *p*-values of specific cancer types using Fisher’s method. Enrichment analysis of functional categories was performed based on gene ontology (GO) annotations obtained from msigdb v5.2 C5 (http://software.broadinstitute.org/gsea/msigdb) with significance assessment by Fisher’s exact test (Additional file 2: Table S2). Among several enriched GO categories that were connected with the biological motives proliferation and immunology the largest of these, ‘cell cycle’ (GO:0007049) and ‘immune system process’ (GO:0002376), were utilized for annotation of the 75 genes. Statistics and graphics generation were performed using the programming language R. Everywhere, *p*-values < 0.05 were considered significant.

## Results

**Fig. 1:**
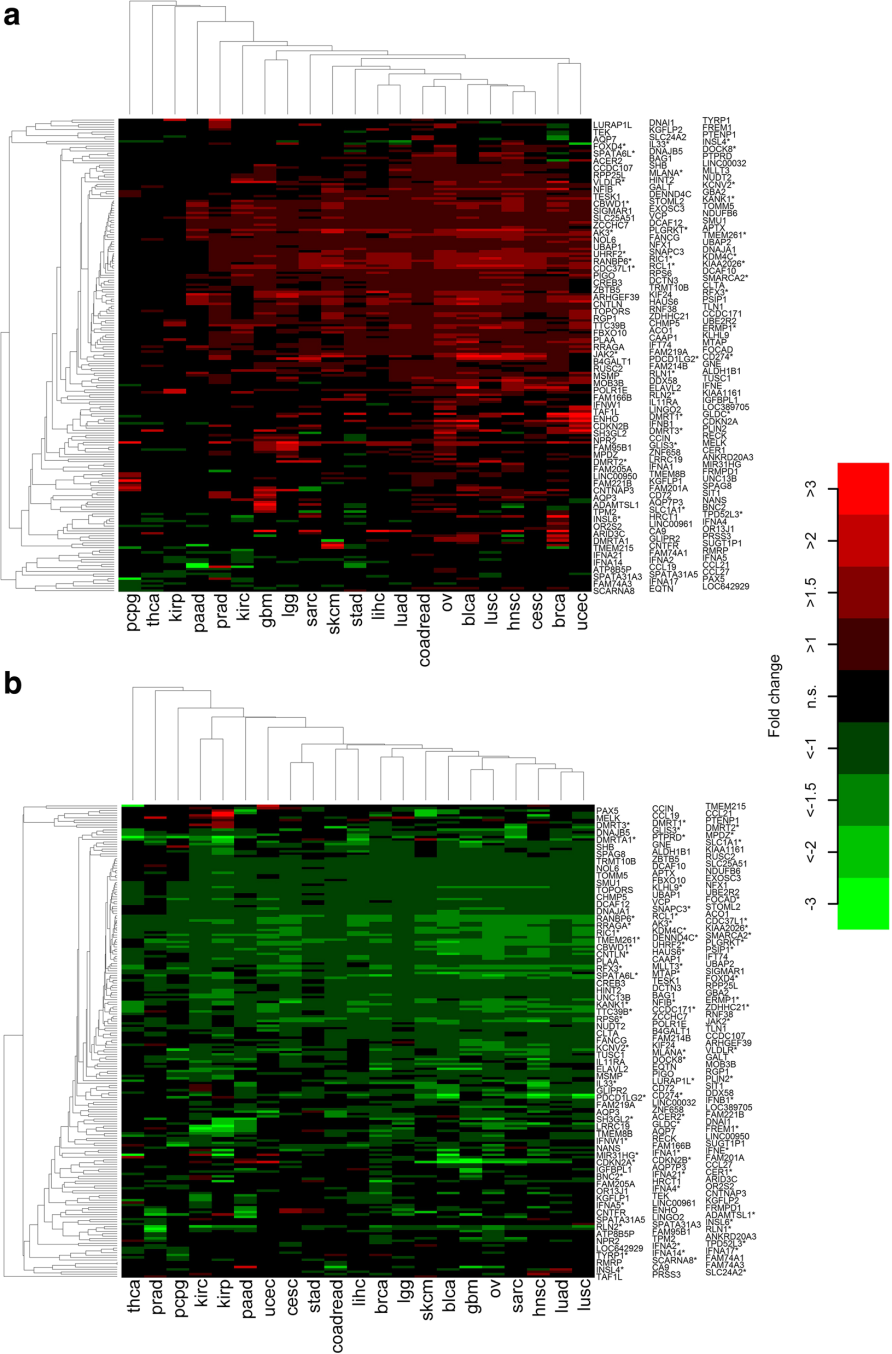
Shaping of mRNA expression of chromosome 9p by copy number alterations in chromosome 9p including *PD-L1*. **a** Heatmap showing mRNA fold changes tumors with and without *PD-L1* gain. **b** Heatmap showing mRNA fold changes of tumors with and without *PD-L1* loss. Significant (*p* < 0.05) changes are highlighted as colored boxes. Genes belonging to recurrently amplified or deleted regions (as identified in [8]) are marked by a star

Comparing tumors with and without *PD-L1* CNG in 22 cancer types, we found significant mRNA expression changes of *PD-L1* in 11 cancer types, of *PD-L2* in 9 cancer types and of *JAK2* in 15 cancer types (Table 1). Some of the detected *PD-L1* mRNA expression changes were very high such as in lung squamous cell cancer (fold change = 4.9), bladder cancer (fold change = 4.1), head and neck cancer (fold change = 2.9), cervical cancer (fold change = 2.7) and cutaneous melanoma (fold change = 2.2). Furthermore, we explored the global mRNA expression pattern of chromosome 9p using hierarchical clustering and heatmap visualization (Fig. 1). In the 11 cancer types with significant *PD-L1* mRNA expression changes, between 124 to 342 genes (15% to 42%) located on chromosome 9p were found to be differentially expressed. Comparing tumors with and without *PD-L1* loss, we identified 14 cancer types with significant *PD-L1* mRNA expression changes and 167 to 415 genes (20% to 51%) with modulated mRNA expression. Genes showing frequent up-regulation in tumors with *PD-L1* gains included *PD-L1*, *PD-L2*, *JAK2,* and *B4GALT1*. Genes showing frequent down-regulation in tumors with *PD-L1* losses included *PD-L1*, *PD-L2* and *IFNB1*.

**Table 1:** Analysis of differential mRNA expression of *PD-L1*, *PD-L2* and *JAK2* in tumors with *PD-L1* CNG compared to tumors without *PD-L1* CNG

Cancer type	PD-L1		PD-L2		JAK2	
	fold change	p	fold change	p	fold change	*p*
Lusc	**4.9**	**1.60E-27**	**2.3**	**3.80E-13**	**1.8**	**3.70E-14**
Blca	**4.1**	**7.70E-14**	**2.6**	**2.80E-06**	**2**	**3.60E-14**
Hnsc	**2.9**	**1.30E-16**	**2.5**	**1.20E-14**	**2.1**	**7.10E-19**
cesc	**2.7**	**2.50E-08**	**2**	**9.00E-05**	**1.9**	**4.90E-07**
skcm	**2.2**	**0.0073**	1.5	0.12	**2**	**0.00026**
paad	2	0.14	1.6	0.5	1.3	0.23
sarc	**1.9**	**0.0028**	**2.2**	**0.00013**	**1.4**	**0.00042**
luad	**1.8**	**0.00078**	1.2	0.12	**1.4**	**7.60E-05**
prad	**1.8**	**9.00E-04**	**1.6**	**0.002**	**1.4**	**0.00017**
stad	**1.8**	**0.0026**	1	0.79	**1.4**	**0.0061**
kirc	1.7	0.18	1	0.98	1.2	0.15
ov	**1.7**	**5.20E-07**	**1.3**	**0.034**	**1.6**	**4.40E-12**
kirp	1.6	0.44	1.1	0.87	1.3	0.45
thca	1.5	0.45	1.4	0.3	1.2	0.4
gbm	1.4	0.34	1.6	0.1	**1.3**	**0.014**
ucec	1.4	0.17	1.2	0.51	1.2	0.28
brca	**1.3**	**0.016**	**1.3**	**0.002**	**1.3**	**2.40E-05**
lihc	1.2	0.52	-1	0.91	**1.5**	**0.0083**
coadread	1.1	0.62	1.1	0.59	**1.3**	**0.00038**
lgg	-1	0.88	**2.5**	**0.004**	**1.4**	**0.00027**
pcpg	−2.6	0.27	−1.3	0.13	−1.2	0.48

Significant changes (*p* < 0.05) are highlighted

Investigated cancer types: Bladder Urothelial Carcinoma (BLCA), Breast invasive carcinoma (BRCA), Cervical squamous cell carcinoma and endocervical adenocarcinoma (CESC), Colorectal adenocarcinoma (COADREAD), Glioblastoma multiforme (GBM), Head and Neck squamous cell carcinoma (HNSC), Kidney renal clear cell carcinoma (KIRC), Kidney Renal Papillary Cell Carcinoma (KIRP), Brain Lower Grade Glioma (LGG), Liver hepatocellular carcinoma (LIHC), Lung adenocarcinoma (LUAD), Lung squamous cell carcinoma (LUSC), Ovarian serous cystadenocarcinoma (OV), pancreatic adenocarcinoma (PAAD), Pheochromocytoma and Paraganglioma (PCPG), Prostate Adenocarcinoma (PRAD), Sarcoma (SARC), Skin Cutaneous Melanoma (SKCM), Stomach adenocarcinoma (STAD), Papillary Thyroid Carcinoma (THCA), and Uterine Corpus Endometrial Carcinoma (UCEC)

Bold data indicate significance (*p* < 0.05)

**Fig. 2:**
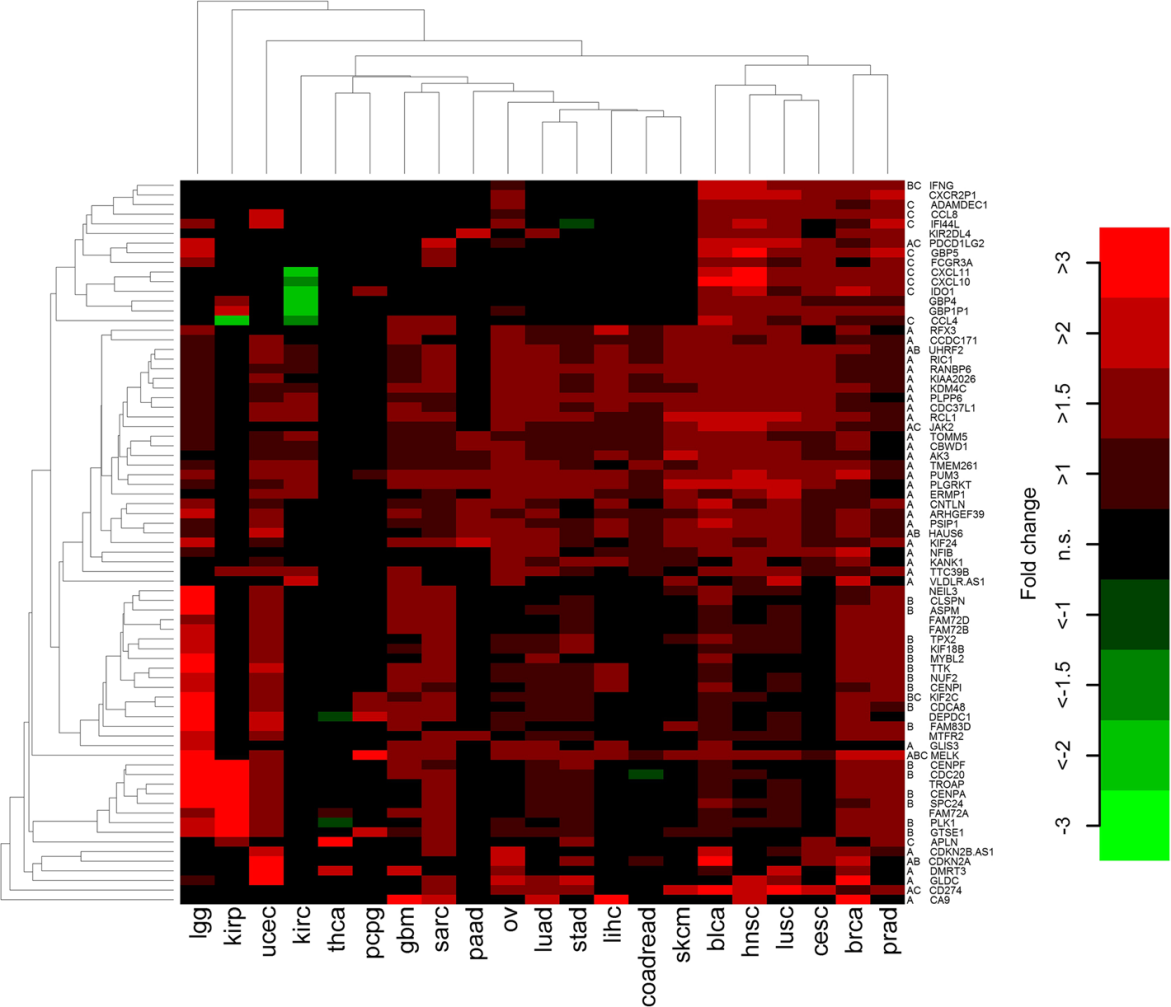
Heatmap of the top list of 75 strongly and recurrent differentially expressed genes (as defined in methods) between tumors with and without *PD-L1* CNGs. Significant (*p* < 0.05) changes are highlighted as colored boxes. The genes are annotated to the following three groups: A = gene located on chromosome 9p, B = gene plays a role in cell cycle regulation, C = gene is implicated in immune system processes

Taking a more comprehensive approach, we analyzed the setting ‘chromosome 9p gain’ in a genome-wide context and identified 75 top genes that were strongly and recurrently up-regulated (Fig. 2 and Additional file 1: Table S1). In a pan-cancer analysis and after Bonferroni correction for 22,849 genes, 74 of these were significantly associated with 9p gain status. While 36 strongly up-regulated genes (48%) were located on chromosome 9p (including *PD-L1*, *PD-L2* and *JAK2*), the remaining 39 genes (52%) were located in other genomic regions. In particular, 21 genes were located in the core amplified region in chromosome 9p24 that we described in [8]. Twenty-two strongly up-regulated genes belonged to the GO category ‘cell cycle’ (*ASPM*, *CDC20*, *CDCA8*, *CDKN2A*, *CENPA*, *CENPF*, *CENPI*, *CLSPN*, *FAM83D*, *GTSE1*, *HAUS6*, *IFNG*, *KIF18B*, *KIF2C*, *MELK*, *MYBL2*, *NUF2*, *PLK1, SPC24*, *TPX2*, *TTK*, *UHRF2*). The vast majority of these genes play a role in the coordinated regulation of microtubule assembly and chromosomal segregation during mitosis. Sixteen strongly up-regulated genes belonged to the GO category ‘immune system process’ (*ADAMDEC1*, *APLN*, *CCL4*, *CCL8*, *CXCL10*, *CXCL11*, *FCGR3A*, *GBP5*, *IDO1*, *IFI44L*, *IFNG*, *JAK2*, KIF2C, *MELK*, *PD-L1*, *PD-L2*). Most of these genes encode cytokines and chemokines but also link immune response with tumor metabolism, such as *IDO1* encoding indoleamine-2,3-dioxygenase 1. Enrichment of the both GO categories was highly significant (cell cycle: *p* = 2.5E-10, immune system process: *p* = 0.00096). Interestingly, as shown in Fig. 2, upregulation of immune system-related genes was particularly enriched in prostate adenocarcinoma, breast cancer, cervical cancer, squamous cell lung cancer, head and neck cancer and bladder cancer, while genes regulating cellular proliferation were strongly expressed in glioma, papillary renal cell carcinoma and uterine corpus endometrial carcinoma well as in breast cancer, prostate cancer, sarcomas, and glioblastomas. Higher expression levels of genes on 9p were observed across most cancer types except for papillary thyroid carcinoma as well as pheochromocytoma and paraganglioma.

## Discussion

Functional genomics analysis of *PD-L1* CNG tumors revealed strong and recurrent mRNA expression changes of genes within and outside chromosome 9p. Before, we showed that approximately half of the *PD-L1* CNGs in the major cancer types occur together with amplification of chromosome 9p or the entire chromosome 9 (non-focal gains), while the other half of *PD-L1* CNGs frequently co-occurs with gain of a 38-gene core amplified region located in chromosome 9p24 (focal gains) [8]. Here were analyzed focal and non-focal CNGs together and identified 75 genes (21 in located the core amplified region) that were strongly and recurrently up-regulated in *PD-L1* CNG tumors. The gene set was enriched for the biological motives ‘cell cycle’ and ‘immune system process’ and included known drivers of tumor growth and drug targets.

A recent study showed that *PD-L1* CNG is a powerful predictive biomarker in patients with relapsed or refractory Hodgkin lymphoma who were treated with nivolumab [3]. In keeping with these data, we recently demonstrated that *PD-L1* amplification accompanied by overexpression can also be used to identify patients with solid tumors who benefit from *PD-1* blockade [4]. These data support a view that *PD-L1* CNGs could help to predict response to checkpoint inhibitors in solid tumors. Testing for *PD-L1* amplifications could be easily implemented in a routine setting using in situ hybridization based assays such as FISH or using targeted DNA sequencing [11]. However, prospective clinical trials and retrospective-prospective analyses of existing clinical trial tissue cohorts are warranted to get a comprehensive picture on the clinical utility of *PD-L1* CNGs in solid cancer types.

Copy number and gene expression analyses uncovered additional potential drug targets, such as amplified and overexpressed *JAK2* (Table 1), which has been implicated in the regulation of *PD-L1* expression [12] and appears to play a role in tumor growth and resistance to chemotherapy [13]. *B4GALT1* encoding β-1, 4-Galactosyltransferase is also frequently co-amplified and overexpressed, and has been demonstrated to mediate multidrug resistance in leukemia cell lines [14]. *PD-L2*, the gene next to *PD-L2* located about 40 kbp toward the centromere, has been demonstrated to be always co-amplified and co-deleted with *PD-L1* across major cancer types [8]. Consistent with this result, we here found the two genes to be co-regulated on transcriptional level in many cancer types. *PD-L2* is a second ligand for *PD-L1* inhibiting T cell activation [15] and has recently been demonstrated to be associcated with clinical outcome in head and neck cancer independent of *PD-L1* [16].

Taking a broader view, our analysis identified two gene expression programs that were significantly associated with cancers harboring 9p gains: regulation of cell cycle and proliferation as well as modulating and fine tuning of immune cell response. As expected, the latter comprised *PD-L1*, *PD-L2,* and *JAK2* already discussed above. Additionally, however, we noted a specific set of genes encoding chemokines and cytokines, namely *CCL4*, *CCL8*, *CXCL10*, *CXCL11*, *IFI44L*, *IDO1*, and *IFNG* that were upregulated in 9p CNG cancers. Chemokines and cytokines are well known to guide macrophages, and T-cells including CD8-positive T-lymphocytes to the tumor microenvironment and are associated with outcome [17, 18]. As the RNA-seq data analyzed here were derived from the entire tumor including its stromal and inflammatory components we were unable to discern whether upregulated expression levels stem directly from tumor cells or adjacent stromal cells. However, these data indicate that cancers with 9p gains involving *PD-L1* are associated with a ‘hot microenvironment’ attracting effector cells of the immune system that could eliminate tumor cells as soon as checkpoint blockade is in place and counteracts the anergic state. On the other hand, chemokines are known to play a role in metastatic spread of tumor cells as they may express CC and CXC chemokine receptors [19–21]. Together with *PD-L1* CNG that hamper successful immune attack due to increased *PD-L1* protein levels, chemokine secretion could drive tumor survival by supporting local or distant spread. Similarly, while *IFNG* exerts clear anti-tumorigenic functions it appears to be also implicated in tumor progression in some settings [22, 23]. Interestingly, among the upregulated genes *IDO1* encoding indoleamine-2,3-dioxygenase 1 was identified. This enzyme, whose expression can be induced by *IFNG* [24], is centrally involved in the degradation of tryptophan in tumor cells whose depletion eventually results in cell cycle arrest and apoptosis of T-cells [25–28], thus possibly supporting *PD-L1* CNG tumors to escape immune surveillance. Conversely, this *IDO1*-mediated mechanism of immune suppression can be exploited therapeutically by inhibition of the catalytic activity of *IDO1* [29] suggesting that dual inhibition of both *PD-1* and *IDO1* could be a promising approach to enhance efficacy of checkpoint blockade. Several studies investigating dual *IDO1*-*PD1* blockade in solid tumor types are underway and preliminary results are encouraging [30, 31].

The second expression program that we identified in cancers with 9p gains includes genes involved in formation of microtubules, chromosome segregation and governing mitosis. For example, *CDC20* encoding cell-division cycle protein 20 homologue plays a role in chromosome separation and transition to G1 phase after mitosis, and may be a suitable drug target [32, 33]. Similarly *PLK1*, which is well known to control metaphase to anaphase transition and mitotic exit, is upregulated in these cancers and pre-clinical as well as clinical data collectively suggest that *PLK1* inhibition can be a promising strategy in cancer therapy [34–36]. Recently, overexpression of *TTK*, another checkpoint in mitosis [37], was reported to occur in triple negative breast cancers [38], which frequently harbor *PD-L1* amplifications [2, 4]. In line with this finding, our analysis showed upregulation of *TTK* across many cancers that harbor CNG of 9p involving *PD-L1*. *MELK* encoding maternal embryonic leucine zipper kinase appears to play a role in governing proliferation and conferring anti-apoptotic properties to tumor cells. With the recent development of inhibitors [39], additional *MELK* inhibition may also be a therapeutic option in patients with *PD-L1* amplified cancers [40].

On a genome-wide scale, recurrent patterns of copy number alterations have been discovered across patients and cancer types suggesting their shaping by selective pressures and roles in tumor genesis and tumor growth [41, 42]. However, the effects of these, in particular of small gene dosage alterations including chromosome or chromosome arm gains or losses, on the phenotypic level are incompletely understood. In a recent pan-cancer analysis, a copy number alteration signature has been linked up-regulation of glycolysis [43], while chromosome 8p loss in breast cancer has been linked to alterations in lipid metabolism [44]. Here, we demonstrated a link of 9p gains to the regulation of proliferation and immune response. Our observations support a view of copy number alterations as hard-wired genetic changes that maintain cancer hallmarks including ‘self-sufficiency in growth’ and ‘evasion from the immune system’ from Hanahan’s and Weinberg’s list [45].

## Conclusions

In summary, we identified a gene signature that is upregulated in *PD-L1* CNG tumors across many cancer types. The data contribute to understanding the biology of these cancers and highlight additional vulnerabilities that might be therapeutically exploitable thus possibly sustaining or enhancing efficacy of checkpoint blockers.

## Additional files

Additional file 1: Table S1. Gene list of 75 top up-regulated genes identified in a genome-wide transcriptome analysis across 22 cancer types. The list was obtained by filtering for genes harboring significant and at least 1.5-fold enhanced mRNA expression in tumors with PD-L1 gains across at least 6 cancer types. Pan-cancer *p*-values were calculated using Fisher’s method (* = significant, ** = significant after Bonferroni correction for 22,849 genes). The set of 75 genes was enriched for the chromosomal regions 9p13 to 9p24 (36 genes) as well as the biological processes ‘cell cycle’ (22 genes) and ‘immune system process’ (16 genes). (XLSX 34 kb)

Additional file 2: Table S2. Enrichment analysis of GO categories in the 75-gene list. (XLSX 240 kb)

## Abbreviations

CNG: Copy number gain
JAK2: Janus kinase 2
PD − 1: Programmed cell death protein 1 (PDCD1)
PD-L1: Programmed cell death 1 ligand 1 (CD274)
PD-L2: Programmed cell death 1 ligand 2 (PDCD1LG2)
TCGA: The Cancer Genome Atlas

## References

[1] Pitt JM, Vetizou M, Daillere R, Roberti MP, Yamazaki T, Routy B, Lepage P, Boneca IG, Chamaillard M, Kroemer G (2016). Resistance mechanisms to immune-checkpoint blockade in cancer: tumor-intrinsic and -extrinsic factors. Immunity.

[2] Barrett MT, Anderson KS, Lenkiewicz E, Andreozzi M, Cunliffe HE, Klassen CL, Dueck AC, McCullough AE, Reddy SK, Ramanathan RK (2015). Genomic amplification of 9p24.1 targeting JAK2, PD-L1, and PD-L2 is enriched in high-risk triple negative breast cancer. Oncotarget.

[3] Ansell SM, Lesokhin AM, Borrello I, Halwani A, Scott EC, Gutierrez M, Schuster SJ, Millenson MM, Cattry D, Freeman GJ (2015). PD-1 blockade with nivolumab in relapsed or refractory Hodgkin's lymphoma. N Engl J Med.

[4] Groschel S, Bommer M, Hutter B, Budczies J, Bonekamp D, Heining C, Horak P, Frohlich M, Uhrig S, Hubschmann D (2016). Integration of genomics and histology revises diagnosis and enables effective therapy of refractory cancer of unknown primary with PDL1 amplification. Cold Spring Harb Mol Case Stud.

[5] Ikeda S, Okamoto T, Okano S, Umemoto Y, Tagawa T, Morodomi Y, Kohno M, Shimamatsu S, Kitahara H, Suzuki Y (2016). PD-L1 is Upregulated by simultaneous amplification of the PD-L1 and JAK2 genes in non-small cell lung cancer. J Thorac Oncol.

[6] George J, Saito M, Tsuta K, Iwakawa R, Shiraishi K, Scheel AH, Uchida S, Watanabe SI, Nishikawa R, Noguchi M (2017). Genomic amplification of CD274 (PD-L1) in small-cell lung cancer. Clin Cancer Res.

[7] Cancer Genome Atlas Research (2014). N: comprehensive molecular characterization of gastric adenocarcinoma. Nature.

[8] Budczies J, Bockmayr M, Denkert C, Klauschen F, Groschel S, Darb-Esfahani S, Pfarr N, Leichsenring J, Onozato ML, Lennerz JK (2016). Pan-Cancer analysis of copy number changes in programmed death-ligand 1 (PD-L1, CD274) - associations with gene expression, mutational load, and survival. Genes Chromosomes Cancer.

[9] Cerami E, Gao J, Dogrusoz U, Gross BE, Sumer SO, Aksoy BA, Jacobsen A, Byrne CJ, Heuer ML, Larsson E (2012). The cBio cancer genomics portal: an open platform for exploring multidimensional cancer genomics data. Cancer Discov.

[10] Mermel CH, Schumacher SE, Hill B, Meyerson ML, Beroukhim R, Getz G (2011). GISTIC2.0 facilitates sensitive and confident localization of the targets of focal somatic copy-number alteration in human cancers. Genome Biol.

[11] Budczies J, Pfarr N, Stenzinger A, Treue D, Endris V, Ismaeel F, Bangemann N, Blohmer JU, Dietel M, Loibl S (2016). Ioncopy: a novel method for calling copy number alterations in amplicon sequencing data including significance assessment. Oncotarget.

[12] Bellucci R, Martin A, Bommarito D, Wang K, Hansen SH, Freeman GJ, Ritz J (2015). Interferon-gamma-induced activation of JAK1 and JAK2 suppresses tumor cell susceptibility to NK cells through upregulation of PD-L1 expression. Oncoimmunology.

[13] Balko JM, Schwarz LJ, Luo N, Estrada MV, Giltnane JM, Davila-Gonzalez D, Wang K, Sanchez V, Dean PT, Combs SE (2016). Triple-negative breast cancers with amplification of JAK2 at the 9p24 locus demonstrate JAK2-specific dependence. Sci Transl Med.

[14] Zhou H, Ma H, Wei W, Ji D, Song X, Sun J, Zhang J, Jia L (2013). B4GALT family mediates the multidrug resistance of human leukemia cells by regulating the hedgehog pathway and the expression of p-glycoprotein and multidrug resistance-associated protein 1. Cell Death Dis.

[15] Latchman Y, Wood CR, Chernova T, Chaudhary D, Borde M, Chernova I, Iwai Y, Long AJ, Brown JA, Nunes R (2001). PD-L2 is a second ligand for PD-1 and inhibits T cell activation. Nat Immunol.

[16] Yearley JH, Gibson C, Yu N, Moon C, Murphy E, Juco J, Lunceford J, Cheng J, Chow LQM, Seiwert TY, et al. PD-L2 Expression in Human Tumors: Relevance to Anti-PD-1 Therapy in Cancer. Clin Cancer Res 2017, 23(12):3158–67 10.1158/1078-0432.CCR-16-1761 28619999

[17] Balkwill F (2004). Cancer and the chemokine network. Nat Rev Cancer.

[18] Galon J, Angell HK, Bedognetti D, Marincola FM (2013). The continuum of cancer immunosurveillance: prognostic, predictive, and mechanistic signatures. Immunity.

[19] Farmaki E, Chatzistamou I, Kaza V, Kiaris H (2016). A CCL8 gradient drives breast cancer cell dissemination. Oncogene.

[20] Barbai T, Fejos Z, Puskas LG, Timar J, Raso E (2015). The importance of microenvironment: the role of CCL8 in metastasis formation of melanoma. Oncotarget.

[21] Liu M, Guo S, Stiles JK (2011). The emerging role of CXCL10 in cancer (Review). Oncol Lett.

[22] Ikeda H, Old LJ, Schreiber RD (2002). The roles of IFN gamma in protection against tumor development and cancer immunoediting. Cytokine Growth Factor Rev.

[23] Zaidi MR, Merlino G (2011). The two faces of interferon-gamma in cancer. Clin Cancer Res.

[24] Takikawa O, Habara-Ohkubo A, Yoshida R (1990). IFN-gamma is the inducer of indoleamine 2,3-dioxygenase in allografted tumor cells undergoing rejection. J Immunol.

[25] Muller AJ, DuHadaway JB, Donover PS, Sutanto-Ward E, Prendergast GC (2005). Inhibition of indoleamine 2,3-dioxygenase, an immunoregulatory target of the cancer suppression gene Bin1, potentiates cancer chemotherapy. Nat Med.

[26] Munn DH, Mellor AL (2007). Indoleamine 2,3-dioxygenase and tumor-induced tolerance. J Clin Invest.

[27] Uyttenhove C, Pilotte L, Theate I, Stroobant V, Colau D, Parmentier N, Boon T, Van den Eynde BJ (2003). Evidence for a tumoral immune resistance mechanism based on tryptophan degradation by indoleamine 2,3-dioxygenase. Nat Med.

[28] Platten M, von Knebel Doeberitz N, Oezen I, Wick W, Ochs K (2014). Cancer immunotherapy by targeting IDO1/TDO and their downstream effectors. Front Immunol.

[29] Zhai L, Spranger S, Binder DC, Gritsina G, Lauing KL, Giles FJ, Wainwright DA (2015). Molecular pathways: targeting IDO1 and other tryptophan Dioxygenases for cancer immunotherapy. Clin Cancer Res.

[30] Ott PA, Hodi FS, Kaufman HL, Wigginton JM, Wolchok JD (2017). Combination immunotherapy: a road map. J Immunother Cancer.

[31] Epacadostat Shows Value in Two SCCHN Trials. Cancer Discov 2017, 7(9):OF2. https://www.ncbi.nlm.nih.gov/pubmed/28760910. 10.1158/2159-8290.CD-NB2017-100 28760910

[32] Kapanidou M, Curtis NL, Bolanos-Garcia VM (2017). Cdc20: at the crossroads between chromosome segregation and mitotic exit. Trends Biochem Sci.

[33] Kidokoro T, Tanikawa C, Furukawa Y, Katagiri T, Nakamura Y, Matsuda K (2008). CDC20, a potential cancer therapeutic target, is negatively regulated by p53. Oncogene.

[34] Strebhardt K, Ullrich A (2006). Targeting polo-like kinase 1 for cancer therapy. Nat Rev Cancer.

[35] Eckerdt F, Yuan J, Strebhardt K (2005). Polo-like kinases and oncogenesis. Oncogene.

[36] Yim H (2013). Current clinical trials with polo-like kinase 1 inhibitors in solid tumors. Anti-Cancer Drugs.

[37] Daniel J, Coulter J, Woo JH, Wilsbach K, Gabrielson E (2011). High levels of the Mps1 checkpoint protein are protective of aneuploidy in breast cancer cells. Proc Natl Acad Sci U S A.

[38] Maire V, Baldeyron C, Richardson M, Tesson B, Vincent-Salomon A, Gravier E, Marty-Prouvost B, De Koning L, Rigaill G, Dumont A (2013). TTK/hMPS1 is an attractive therapeutic target for triple-negative breast cancer. PLoS One.

[39] Chung S, Kijima K, Kudo A, Fujisawa Y, Harada Y, Taira A, Takamatsu N, Miyamoto T, Matsuo Y, Nakamura Y (2016). Preclinical evaluation of biomarkers associated with antitumor activity of MELK inhibitor. Oncotarget.

[40] Gray D, Jubb AM, Hogue D, Dowd P, Kljavin N, Yi S, Bai W, Frantz G, Zhang Z, Koeppen H (2005). Maternal embryonic leucine zipper kinase/murine protein serine-threonine kinase 38 is a promising therapeutic target for multiple cancers. Cancer Res.

[41] Beroukhim R, Mermel CH, Porter D, Wei G, Raychaudhuri S, Donovan J, Barretina J, Boehm JS, Dobson J, Urashima M (2010). The landscape of somatic copy-number alteration across human cancers. Nature.

[42] Zack TI, Schumacher SE, Carter SL, Cherniack AD, Saksena G, Tabak B, Lawrence MS, Zhsng CZ, Wala J, Mermel CH (2013). Pan-Cancer patterns of somatic copy number alteration. Nat Genet.

[43] Graham NA, Minasyan A, Lomova A, Cass A, Balanis NG, Friedman M, Chan S, Zhao S, Delgado A, Go J (2017). Recurrent patterns of DNA copy number alterations in tumors reflect metabolic selection pressures. Mol Syst Biol.

[44] Cai Y, Crowther J, Pastor T, Abbasi Asbagh L, Baietti MF, De Troyer M, Vazquez I, Talebi A, Renzi F, Dehairs J (2016). Loss of chromosome 8p governs tumor progression and drug response by altering lipid metabolism. Cancer Cell.

[45] Hanahan D, Weinberg RA (2011). Hallmarks of cancer: the next generation. Cell.

